# An Essay on Croup

**Published:** 1856-08

**Authors:** 


### *An Essay on Croup.* By S. M. Bemiss, M. D.

Croup is a disease of such general prevalence and fatality, that but little apology is necessary for frequently calling the attention of medical men to its consideration. It should perhaps rather be held among members of the profession a matter of duty to bring forward, each the result of his observation, so that, by united contributions, we may establish correct principles of pathology, and such appropriate treatment of the disease, as to diminish, at least to some extent, the humiliating ratio of mortality heretofore marking its progress. The chief obstacle to the investigation of this subject, has been difference in diagnosis, or more properly perhaps, difference in the use of the term croup. Some writers apply the designation in its original sense to a symptom merely, and include in their descriptions various pathological conditions attended by stridulous respiration; others regard the presence of false membrane in the larynx or trachea necessary to its definition. This difference has led to a great deal of confusion and apparent discrepancy among authors, and renders it necessary to examine medical observations closely, to ascertain whether they relate to true or false croup. For the remedy of this evil, some authors have advised an abandonment of the word croup, and numerous scientific terms have been proposed as substitutes. But, as stated by Roger Collard, none of them convey to the mind but a part of the phenomena of the disease; and as the term is in general use I shall adhere to it, premising that whenever I employ the word without other specification, I have reference only to true pseudo-membranous croup. In the course of the article I shall take occasion to notice those affections which, from resemblance of symptoms or supposed analogy of character, have been associated with membranous croup, and shall then discuss the propriety of classing them under the same denomination.

The mortuary lists of various countries exhibit a striking uniformity in the rate of mortality from croup. This observation will even apply to countries subject to climatic influences as different as England and Kentucky. In the former country, the deaths for five successive years amounted annually to about one in every 3500 of the whole population; in Kentucky, for three successive years the annual report of deaths under this head, was about one in 3000 of the white population, and in something less than 2000 of the negro race. Massachusetts and New Jersey exhibit a ratio of over one in 4000, and Connecticut less than one in the same number of their respective population for the year 1854. If we take into account large urban populations alone, this relative ratio is almost always greatly increased: Boston, New York, and Philadelphia lose annually about one in 1000 of their population from this cause. These estimates are in many instances only approximative, but they are believed to fall below rather than to exceed the truth.

The increase of population since 1850 impairs to some extent the correctness of statistics based upon the census of that year.

*Symptoms.*—The symptoms of croup are divided into those of invasion; those of development or confirmation; and those of collapse or suffocation. The symptoms of the first stage are, in a majority of instances, those of ordinary catarrh. The child complains of light chills; of malaise, probably of its head, or throat; there is more or less fever, the cheeks are flushed, the eyes watery, the discharge from the nares, sometimes unusually transparent and ropy, is increased. The voice and cough are often somewhat hoarse, but as the child frequently pursues its usual pastimes without any symptoms of such gravity as to demand immediate interference, it seldom occurs that the apprehensions of its friends are excited, and the physician is rarely summoned during this stage. These precursory symptoms may continue for a longer or shorter period, varying from ten or twelve days to a few hours. They may even be wanting, according to some observers, though Ryland says the precursory catarrh exists 'probably in all cases, but often passes unnoticed on account of the extreme youth of the subjects of this disease, and the undistinguishing character of the symptoms themselves.'

The symptoms of the second stage date from the accession of the characteristic cough and respiration. The lesions to which these peculiar modifications are due, undoubtedly have their origin during the first stage, and may precede the actual symptoms of development for a longer or shorter period, in measure with the rapidity of march of the disease. These lesions are probably at first more or less extensive catarrhal irritations or hyperemia of the mucous membrane, merging itself into inflammation (Hasse,) and pouring out the material of the false membrane. As this concrete exudation, and its attendant serous infiltration encroach upon the larynx, they impede its functions, and produce alterations of respiration, cough, and voice pathognomonic of the disease. The earliest change in the respiration is, according to Dr. Ware, a mere exaltation of the act, denoted by a little more than usual exertion of the voluntary respiratory muscles, a 'slight dilatation of the nostrils, and a little whiz or buzz accompanying the passage of air through the rima glottidis.' As the larynx, and especially the glottidean chink, are invaded, the respiration becomes laborious, stridulous, and sibilant. Drs. Vauthier and Blache have compared the sounds of the respiration to the movement of a saw through soft stone, and there is justice in the comparison. The respiratory act is always hurried; in Vauthier's cases it averages from 36 to 40 per minute; the greatest number was 56. 'A symptom of the greatest practical value in regard to the respiration in croup is the sudden and paroxysmal exacerbations of the dispnœa which constantly exist.—For instance, we witness a certain degree of obstruction to the respiration, which appears to be mechanical, and is continuous; the violent aggravations of dyspnœa, not persistent in their character, and evidently attributable to spasm. These paroxysms have a tendency to periodicity, and recur more often at night with obvious remissions towards morning. They may however be induced by any excitement or provocation, by deglutition, coughing, or attempts of the physician to make a close examination.'*

It is impossible to describe the characteristic cough of croup so as to con-

*Vauthier, Archives Gen. de Med. Mar. 1843

vey a correct idea of its sound to the inexperienced, while to the experienced, description is unnecessary, for it is so peculiar, and often connected with such painful associations, that those who have heard it seldom fail to recognize it again. It very often first announces itself at night, and is, at the incursion, usually loud, metallic and harsh; but as the exudation progresses along the larynx, and the vocal chords become veiled, it loses the clangous resonance and sounds as though it were stuffed and pent up in the trachea (Guersent). During the dyspnœal fits it undergoes a corresponding aggravation, becoming tighter and more embarrassed.

The voice at the debut of croup is generally cracked and hoarse, and as the vocal chords become involved, it is whispering, feeble, and often completely lost. Aphonation is almost always reluctantly performed, and the child often endeavors to express its wants by signs rather than attempt to speak, the repugnance being doubtless oftentimes due to the pain attending the effort. Louis' adult patients complained of a tearing sensation produced in the throat by efforts to speak.*

The confirmed stage of croup is accompanied by more or less strongly pronounced febrile symptoms; the pulse is generally frequent and hard, the tongue red at the tip, loaded and white at the base; the tonsils are frequently swollen, and very often the seat of the fibrinous deposit. The external throat and face may present a puffed appearance; sub-maxillary glands are quite often enlarged, and the line of the larynx and trachea tender to pressure. Deglutition is generally performed without other difficulty than temporary interference with respiration. The skin is sometimes hot and dry, but, in many instances, it is soft and of normal temperature; during the accession of dyspnœa it is frequently bathed in perspiration. The bowels are ordinarily rather inactive; the digestive functions and appetite have, in a majority of my observations, been uninterrupted except by the influence of nauseants.

The third or collapsed stage is marked by indications of failing vitality, such as more or less lividity of the lips or face, flagging or intermittent pulse, constant somnolency, and coldness of the extremities and surface. The voice in this stage is either a faint whisper or entirely lost. The respiration retains its characteristics, but there is more continuous difficulty in its performance; it has often a more strongly marked wheezing sound as of 'air forced by a piston through a dry valve;' and the soft parts of the thorax sink deeply at each inspiration. The cough, dry, feeble, and ineffectual, becomes in most cases less frequent, and occasionally entirely ceases. These symptoms go on increasing in severity until the patient perishes either comatose, or in a violent dyspnœal paroxysm.

The appearance of a child after the full establishment of croup is generally that of inexpressible distress. The dread of impending suffocation is plainly depicted in his countenance, and as the mental faculties are retained to the last, his intelligent watchfulness of the ministrations around his bed, and the gloomy calmness of despair written on his features, form pictures too touching to be readily erased from the memory of the observer. The decubitus is usually dorsal, with the head carried back. The hand is frequently brought to the front of the neck and is sometimes passed into the throat in vain efforts to remove the mechanical obstruction to respiration.

The appearance of the false membrane upon the fauces is a most impor-

---

*Du croup considere chez l'adutte.

tant symptom in croup. It may often be observed at any period of the disease, but should be regarded as a symptom proper to the first stage, since its manifestation is nearly always anterior to the full confirmation of the malady. It is seen in distinct whitish patches, or a continuous sward upon the tonsils, uvula, base of tongue, or any part of the posterior throat susceptible of being brought into view. Dr. Ware found it present in 32 of 33 cases under his observation. Guersent considered it commonly present in the first stage, but often removed by natural processes or local applications, so as not to be visible later in the disease. These facts cannot be too strongly urged upon the attention of physicians, for they teach the importance of carefully inspecting the throat in every case of suspected croup or catarrh brought under their notice.

The expectoration in croup varies greatly in quantity; sometimes there is little or none, sometimes an abundant viscid ropy mucus collects in the fauces, and increases the difficulty of respiration, until it is removed either by swallowing or the act of vomiting. Fragments of false membrane more or less large and irregular in form are occasionally ejected. Towards the latter stage, the expectoration is often muco-puriform in appearance.

*Complications.*—The most frequent complications of croup are pneumonia, bronchitis and the exanthematous disease. Barthez and Rilliet found pneumonia in five-sixths of their cases. Wert also regards it as a common complication, and states that it was double in all the cases in which he noticed its presence.

According to Copeland, 'the extension of the inflammation to the bronchia and lungs, is indicated by unremitting persistence of symptoms, deep suffocative cough, lividity and leaden hue of the countenance, co d clammy perspiration and somnolency.' Recent pathological investigations also indicate that fibrinous concretions of the heart are not uncommon complications.*

The value of auscultation in cronp is very much diminished by the fact that its signs are masked by the sounds of respiration, and that attempts at this method of examination agitate the patient and increase the stridor of his breathing. Mr. Barth was of opinion that a vibratory sound (comme si un voile mobile était agité par l'air) indicated the existence and limits of the false membrane; but it is probable that this sound is connected only with those occasional instances in which a partially detached membranous fragment moves to and fro with the respiratory currents. It is likely that its benefits are limited to the information it conveys of the 'amount of obstruction to the entrance of air into the lungs, and the extent of disease of the air tubes or substance of the lungs, which accompanies it.' (West.)

*Pathological Anatomy.*—The anatomical lesions peculiar to croup relate to the appearance and extent of the exudation, and the condition of the subjacent parts.

The false membrane is of grayish white color, and varies in consistence from that of thick cream to tough leather, (Rokitansky.) It is sometimes disposed in irregular plates, the intervening mucus surface presenting a normal appearance: again it is found covering the whole wall of the larynx and trachea with an unbroken pavement, reaching even to the small bronchial

*London Lancet, vol. 1, 1854.

tubes. Dr. Vauthier obtained from one of his patients so perfect a duplicature of the trachea that it was susceptible of insufflation. Its thickness varies from that of ordinary writing paper to one and a half lines. It is nearly always thickest along the posterior wall of the larynx and trachea. Its most frequent location is the larynx, reaching to the upper part of the trachea.—In 54 autopsies M. Bretonneau found the false membrane terminating in different points of the trachea in 31; penetrating the large bronchia in 16; reaching the terminal bronchia in 7, (Guersent). Dr. Budd conceives that the deposit has a peculiar and unexplained predilection for the right bronchus. The surface of ulcers, of wounds made in the performance of tracheotomy, or any part deprived of the cuticle may occasionally become the seat of exudation. The false membrane has chemical reactions identical with the fibrin of the blood. It has never been observed to be organized, but small points of incipient fibrilation have been found to exist. It is characterized by an early tendency to break down and liquify (Rokitansky), and during this process it sometimes becomes corrosive, and ulcerates the subjacent tissues.

With regard to the state of the structures beneath, West says, 'On removing the false membrane from the trachea, the lining of the tube is seldom found to present any change other than an increase of its vascularity, which, though sometimes very considerable, does not bear any certain relation to the amount of false membrane present.' Rokitansky says the submucous tissues, especially the areolar, appear to be infiltrated. It is worthy of remark that in a majority of dissections of patients dying from croup, the mechanical occlusion of the glottis by either the membrane or its subjacent swelling is not so complete as to account for death.

*Causes.*—Before adverting to those causes which seem to excite attacks of croup, and are immediate in their influence, it will be proper to consider the circumstances of age, sex, and constitution most favorable to its development.

Croup is essentially a disease of childhood; and although no period of life is altogether exempt, it probably finds most victims between the ages of two and four years. Of 27 cases reported by Vauthier, 24 were between those ages. Of thirteen cases of which neighboring physicians have furnished me notes, 9 were between these periods, 2 over five, and 1 under two. Of 10 cases under my own observation, 7 were between two and four, 2 under two, and 1 over five. Of 1401 deaths from this disease in Kentucky during the years 1852, '53, and '54, 1241 were under the age of five.

Most observers agree that males are more liable to croup than females.—Of 1389 cases in Kentucky, 764 were males and 625 females.* It is probable that this seeming law of the disease is set aside by epidemic influence. In the epidemic recorded by Dr. Vauthier, the deaths were 21 females to 14 males. In an epidemic of 6 cases witnessed by Dr. Wible, 4 were females. Of 7 cases attended by Dr. Meigs while croup was epidemic in Philadelphia, 5 were females. The facts, however, with regard to the relative liability of the sexes, in either sporadic or epidemic croup, are not sufficiently numerous to justify any absolute conclusions.

It will require further observations to decide positively the state of constitution most favorable to attacks of croup. Jurine, a very accurate observer, thinks the lymphatic temperament most amenable to its incursions. I am clearly of his opinion, though it is not to be denied that the weight of med-

*Registration Reports of Kentucky for 1852, '53, and '54.

ical authority is against this view. Of the 10 cases under my notice, 6 were undoubtedly of a constitution in which this temperament preponderated. It is an incontestible fact that strong family predispositions exist towards croup. Of the ten cases just referred to, two occurred in one family and two in another, and in one of these families there had been previously two deaths from the same disease. It is well to remark also that no two deaths occurred in the same family within a period of a year, except in one instance; so that no epidemic influence, or simultaneous subjection to exciting causes can be alleged, to contravene the supposition of family diathesis.

One of the most remarkable predisposing causes of croup is early weaning. So many close observers agree upon this point, that we may receive it as an established fact. Of the 10 cases under my observation 5 were children of females, who, from frequent gestation, were obliged to wean their children early. Other conditions, such as enlargement of the tonsils, and the influence of exanthematous or other disease, also predispose to the disease.

The immediate causes of croup, are generally referred to atmospherical conditions. Cold, especially humid cold, is probably the most common exciting cause. The influence of temperature is shown by its greater frequency during the cold months of the year. Of 18 cases occurring in the city of Boston in 1852, 132 were in the first three and last three months of the year. The months showing respectively the highest and lowest number of deaths for three years in Kentucky, were October and May.

The Registration Report of Kentucky for 1854 shows a large increase of the deaths from croup over that of the previous year. The last eight months of 1854 were remarkable for extreme, almost unbroken dryness of the atmosphere, and it is a singular fact, that nearly the entire increase of deaths for the year occurred during its terminal half. This will be more clearly shown by the following teble:

| | Deaths. | | Deaths. |
|---|---|---|---|
| First six months of 1853, | 207 | Last six months of 1853, | 173 |
| " " " " 1854, | 218 | " " " " 1854, | 321 |
| Excess - - - - - - - - | 9 | Excess, - - - - - - - - | 148 |

I shall not attempt any explanation of this seeming contradiction to previous observation, but simply suggest that the continual irritation of the respiratory mucous membrane by particles of floating detritus might tend to induce the catarrhal irritation and hyperemia preliminary to croup. When treating of the pathological condition of the system in croup, I shall refer again to this point.

*Diagnosis.*—The diseases most frequently confounded with croup are catarrh, laryngitis, laryngismus, and diptheritis. I propose to notice these only so far as to point out the resemblance and relations they bear to membranous croup.

1st. *Catarrh.*—The catarrhal or mucous croup of some authors, does not in my opinion deserve to be considered a variety of croup. Congestion about the glottidean chink, and viscid mucus adhering to the walls of the larynx, may give rise to hoarseness and stridulous cough, bearing sufficient analogy to croup to awaken some anxiety; but the diagnosis is not difficult; the symptoms lack the gravity and intensity of those of croup; and with the subsidence of the slight febrile action attending the affection, renewed secretion occurs and relieves the congested membrane. The bronchitic, rather than the croupal nature of the disease determines the diagnosis.

2d. *Laryngitis.*—Laryngitis or inflammatory croup is, in the child, very often mistaken for membranous croup. The diagnosis is generally very difficult, but, in a practical point of view, important. I shall have occasion under a subsequent head to express my views of the difference in the nature of the two affections, and shall here consider only the question of diagnosis. Barthez and Rilliet* remark that 'the diagnosis in certain cases is so difficult that practitioners the most experienced have been and still will often be led into error.' The following is a comparative synopsis of symptoms from these authors:

| Pseudo-Membranous Croup. | Laryngitis. |
|---|---|
| Pseudo-membranous angina often precedes the invasion. | No preceding diptheritic angina. |
| The symptoms of invasion exhibit generally less gravity, the fever is less intense, excitement less marked. | The disease announces itself like the severe phlegmasiæ by very intense fever and great agitation. |
| The raucity of the cough and hoarseness increase progressively. | The cough is very raucous from the onset, the severe hoarseness results in aphony. |
| The dispnœa exhibits itself in violent paroxysms which often leave between them a manifest remission. | The dispnœa exists from the beginning and augments progressively; very rarely attended by suffocative fits. |
| The febrile movement is rarely of a high degree of intensity. | The fever is violent during the whole course of the disease. |
| The intellectual functions remain unaffected. | Delirium and convulsions sometimes occur. |

Laryngitis is attended with more pain, heat, and tenderness in front of the throat. The expectoration is frothy ropy mucus. The only positive points of diagnosis, however, are the appearance of the false membrane upon the fauces, or its expectoration in the one disease and its absence in the other. We have not sufficient statistics to justify any conclusion with regard to the frequency of laryngitis in early life. Under Dr. Ware's observation its relative frequency to croup was as 18 to 22. The average duration of laryngitis in the child is much less than that of croup. Its career, more synochal in type, terminates, if fatal, equally as soon, and if favorable, much sooner than that of croup.

3d. *Laryngismus.*—Laryngismus, or the spasmodic croup of some authors, is more often confounded with membranous croup than any other disease. A large majority of those sudden attacks reported by some writers as violent cases of croup resulting in speedy cure, are doubtless only instances of laryngismus. It is, in its purity, a disorder of the diastaltic spinal system; or, according to Marshall Hall, it is rather a symptom than a disease, and may attach itself to many conditions, and appear in various grades of severity.—It is a frequent attendant upon the process of dentition, and is referable then to irritation of the trifacial nerve. Irritation of the pneumogastric nerve from overfeeding or indigestion may also occasion it. Dr. Ley relates cases

*Maladies des Enfants.

in which it seemed to be occasioned by pressure on the pneumogastric from enlarged bronchial glands. Dr. T. G. Richardson of this city has met with a similar instance. In this latter forms it is, according to Dr. Hall, 'paralytic,' persistent in its recurrences, and fatal. Under almost all other circumstances it ceases simultaneously with the removal of the exciting cause. It is more apt to appear in children of nervous temperament. The attack is liable to come on at night, sometimes recurring only at night, and manifesting occasionally a remarkable tendency to periodicity in its returns. I have notes of a case, recently witnessed, in which the paroxysms recurred within a short period of 7 o'clock, for three successive evenings. The advent of laryngismus is sudden, seldom preceded by catarrh, or any prodrome calculated to attract attention to the respiratory organs. The cough is more hoarse, raucous and sonorous than that of croup. The voice is hoarse and cracked, but never lost. The respiration is frequently very hurried and laborious; it reached 60 in a minute in a case under my observation. In laryngismus the inspiration is difficult, and exspiration comparatively easy; in croup both acts are obstructed. The cyanosis, intermittent pulse, and reverted head are often as strongly marked as in the last stage of membranous croup. During the remissions of membraneous croup, the cough and respiration, though moderated in their intensity, retain their pathognomonic character; while in the intervals of the paroxysms of laryngismus the patient is often almost entirely exempt from indications of disease. There is seldom persistent fever in laryngismus, though it sometimes occurs that an inflammatory element, general or local, is combined with the affection, and gives it a febrile type. Family idiosyncrasy will often assist in determining the diagnosis; if the patient or other members of the family have had laryngismus or infantile convulsions, the presumption will be strong that the present is a convulsive affection. There is no appearance of false membrane upon the fauces in laryngismus, nor expectoration which would justify a belief that such exudation had existed in any part of the tracheal course. Dr. Churchill is of the opinion that the disease may, by neglect, be converted into membranous croup, but the records of medical science are so barren of such instances that we may consider their rare occurrence as simply an accidental blending of the two affections. This malady should, in our opinion, be regarded as a complication, but not a variety of croup.

4th. *Diptheritis, or Secondary Croup.*—This affection possesses so many symptoms in common with idiopathic croup, and so few of striking contrast, that I am confirmed in the opinion that they are true congeners, and should be classed as such. Dr. David Welsh, in an account of diptheritis which appeared in the Ohio Med. and Surg. Journal, May, 1850, gives the following symptoms of invasion: 'The constitutional symptoms were generally vague and deceptive at the outset, there being but little to attract the attention of the careless observer—generally nothing more than apparent lassitude with a dulness of expression and slight tendency to somnolency, and generally moderate febrile excitement and derangement of the secretions; the patients complaining little or none.' The first local manifestation, as in idiopathic croup, is upon the fauces. There first appeared blotches upon the mucous membrane and 'very soon there could be seen concretions forming upon the inflamed surfaces, first in small circumscribed patches of irregular shape, not very dissimilar to patches of curdled milk, of varying shades of color, at other times of a dirty bluish tint. As the inflammation went on, these inspissated concretions spread and coalesced, presenting the appearance of false membranes, sometimes covering the entire pharynx and velum palati.'

Although the point of local development in these two affections is the same, their mode of progression differs somewhat. That of croup, as we have seen, is nearly always from above downwards, while that of diptheritis is very often in different directions. The exudation of diptheritis differs from that of croup in its greater tendency to occur at first in insulated patches, and by its greater aptitude to exhibit itself upon blistered, abraded, or ulcerated surfaces. The esophagus, stomach and vagina are occasionally seats of its development. The exudation is also softer, and undergoes decomposition earlier than that of croup. Diptheritis is a more ataxic condition of disease than croup, and is often attended with fetid breath, diarrhœa, and well marked typhoid indications. The fetor of the breath, and probably the diarrhœa, are due to the putrefaction of the deposit, (Empis). More or less swelling of the submaxillary glands is said to be always present. Diptheritis is epidemic in its tendencies, and sometimes impresses large districts with its influence. It has occasionally appeared to be contagious, but this point is not positively established. The history of the case and prevalent diathesis may serve to establish the diagnosis previous to the invasion of the larynx; after that event, the disease may, for all practical purposes, be regarded as one of the true croup, subject to the same laws of prognosis and treatment.

*Nature.*—The nature of croup is a subject so difficult of explanation, that farther investigations seem to be yet required to settle beyond question some points of its pathology. The idea has long been held by most of the profession, that it is purely a local inflammation, differing only in degree from simple acute laryngitis. Others again have taken the ground that the expression of inflammatory symptoms, either local or general, has not so much agency in determining the presence or absence of false membranes, as its accidental dislodgement from the throat by expectoration. I am not prepared to deny that this latter circumstance may not occasionally prevent the concretion of the exudation, but that it has any just claim to be considered as the sole cause, or even a common cause, of the difference between croup and laryngitis, is, I think, a matter of inference only, and not of proof. I do not think the phenomena of croup will support either of the above conclusions. On the contrary, many of them clearly point to an essential difference in the charactor of these affections—a difference which does not regard the intensity or extent of the inflammation so much as the state of the system at the time of its incursion. Without attempting to discuss this question as fully as its importance demands, I shall trust the safety of my conclusion to one or two points of contrast between croup and laryngitis, and to the opinions of the distinguished pathologists upon whose researches it is based. If with a view to comparison, laryngitis be taken as the type of a local inflammation in a normal state of the constitution, the deviatious of croup from the standard are shown by the following facts: 1st. That traumatic inflammations of the larynx do not result in croup. Barthez and Rilliet quote an exception, it is true, where the child of a druggist died of croup from inhaling the vapor of muriatic acid; but as far as my researches have been made, this is the only case of the kind on record, and I am disposed to regard it as a mere coincidence of cause and predisposition. Scalds of the glottis sometimes give rise to an exudation, but it is confined to the points of primary lesion, and does not, like that of croup, extend continuously over large surfaces of membrane. 2d. Although fibrinous exudation relieves local inflammation, as Rokitansky and others have shown, yet the tendency of croup membrane is to reproduce itself when forcibly removed from a surface; this would not be likely to occur if the disease were purely local. 3d. In croup, exudation upon ulcerated or abraded surfaces are not very uncommon; in laryngitis, they never occur.

These differences in character appear to me to be explicable only by the supposition of a constitutional diathesis in croup, and are, I think, best accounted for by the teachings of the Vienna school of pathology, to which, however, my limits will allow only a brief allusion. Rokitansky contends that croup is but the local manifestation of a constitutional disorder, and supposes this derangement of the constitution to be a deviation from the normal state of the fibrine of the blood. He denominates this condition the 'fibrine crasis,' and supposes the change may be either qualitative or quantitative. Dr. Mauthner of Vienna says: 'that increase of, and qualitative changes in the fibrine of the blood are found to be favorable conditions for the occurrence of croupose products is not to be denied, since it is a clinical fact, that, in many cases, such a state of this fluid precedes the local affection. But our knowledge concerning the blood crasis, being yet very imperfect, it may occur to the most experienced that croupose exudation shall be formed, of whose existence he is ignorant, or only knows when too late.' The causes and nature of this pre-existent crasis in croup look to future observations for their establishment. Dr. Theophilus Thompson* seems to regard super-fibrination of the blood as a cause of fibrinous exudation, and supposes excessive use of animal food likely to produce this state of hyperinosis. If this supposition be correct, it may in part account for the frequency of croup among children who are taken early from the breast, and fed upon diet of this description. It may also account, in part, for the increased prevalence of croup in Kentucky during the latter part of 1854, when from the extreme dry weather, the supply of vegetables was deficient, and a greater resort to animal food necessarily induced. The same causes excited to produce an epidemic of croup under the observation of Dr. Vauthier in Paris during the year 1846–7.† However little we are able to say positively upon this subject, we know that the state of respiration in croup is not favorable to the increase of fibrine, and if its excess in the blood be found to exist in that disease, the inference is very strong that the increase occurred at least prior to the obstructed respiration.

It may be stated then that while catarrhal hyperemia of the throat and larynx may be equally the exciting cause of croup and laryngitis, the pre-existent crasis is the cause that determines the nature of the disease. I would account for its great prevalence among children by reference to the following circumstances: 1st. The maladaptation of diet to the systemic wants of children induces more frequent derangement of the constituents in their circulating fluids. 2d. The greater frequency of catarrhal irritations, as exciting causes. 3d. The greater tenuity of the mucous membrane and the walls of its terminal vessels in children, is more favorable to the exudative process. The converse of this, combined with a greater laxity of the sub-mucous areolar tissue in the adult is more favorable to infiltration beneath (œdema glottidis) than to exudation upon the membrane. 4th. In the adult, the greater

*London Lancet, 2d vol., 1850. †Archives Gen. de Med., Mar. 1848.

calibre of the larynx, and ability to expectorate, favor the expulsion of the exudation prior to its concretion.

With regard to the formation of the false membrane, Rokitansky says, 'from analogy, it is probable that at the commencement of the process a serous fluid is effused, by which the epithelium of the diseased mucous membrane is destroyed, and that the exudation of the plastic matter takes place afterwards.' When the system is in a sthenic condition and the local inflammation not intense, the exudation assimilates, in its power of organization, more to the blood plasma, and its concretion is more perfect.* The converse is equally true, for we observe in diptheritis, where the local inflammation leads to ulceration, as it does not unfrequently, and the state of the system is asthenic, the exudation has a more feeble power of organization, is soft, of a semi-purulent character, and undergoes rapid decomposition. The strong disposition this exudation manifests to pass from the pharyngial to the laryngial surface has not been explained. Empis supposed that exposure to air was more favorable to the exudative process, which, if correct, may serve to account for this preference.† These observations are, I think, important to a knowledge of the nature of croup.

Laryngismus is also an important element in the nature of croup, with which it is always more or less combined, and upon the character of which it stamps, more or less completely, its own impress. The periodic exacerbations of croup, and the aggravation of its symptoms under emotional excitement, are undoubtedly due to this spasmodic complication. The best explanation that can be offered of the tendency of croup to nocturnal announcement and aggravation, is that given by Marshall Hall, and quoted by Dr. Newman. 'Volition has a constant influence over some of the muscular actions, of which we are almost unconscious, and which we only discover by carefully observing the effects of its subtraction. The acts of respiration originating, as they do, in the reflex function of the spinal cord, are nevertheless regulated and rendered equable by the silent but constant influence and agency of volition.'‡ Thus during the withdrawal of volition in sleep, the laryngeal muscles are more amenable to spasmodic influence, and by their irregular contraction produce the dispnœal fits. During the latter stages of croup convulsions occasionally occur, ascribable no doubt in many instances to a poisoned state of the blood from retained carbon.—*Louisville Review.*

---

*West on Diseases of Children, and Carpenter's Human Physiology.
†Archives Gen. de Med. Feb. 1850. ‡Am. Jour. Med. Sciences Jan. 1856.

(To be continued.)

